# Early subtropical forest growth is driven by community mean trait values and functional diversity rather than the abiotic environment

**DOI:** 10.1002/ece3.1604

**Published:** 2015-08-06

**Authors:** Wenzel Kröber, Ying Li, Werner Härdtle, Keping Ma, Bernhard Schmid, Karsten Schmidt, Thomas Scholten, Gunnar Seidler, Goddert von Oheimb, Erik Welk, Christian Wirth, Helge Bruelheide

**Affiliations:** 1 Martin Luther University Halle-Wittenberg, Institute of Biology/Geobotany and Botanical GardenAm Kirchtor 1, D-06108, Halle (Saale), Germany; 2 Faculty of Sustainability, Institute of Ecology, Leuphana University LüneburgScharnhorststr. 1, D-21335, Lüneburg, Germany; 3 Institute of Botany, CAS20 Nanxincun, Xiangshan, Beijing, 100093, China; 4 University of ZurichWinterthurerstrasse 190, CH-8057, Zürich, Switzerland; 5 Physical Geography and Soil Science, University of TübingenRümelinstraße 19-23, D-72070, Tübingen, Germany; 6 Institute of General Ecology and Environmental Protection, Technische Universität DresdenPienner Str. 7, 01737, Tharandt, Germany; 7 University of LeipzigJohannisallee 21–23, D-04103, Leipzig, Germany; 8 German Centre for Integrative Biodiversity Research (iDiv) Halle-Jena-LeipzigDeutscher Platz 5e, D-04103, Leipzig, Germany

**Keywords:** BEF-China, community-weighted mean traits, ecosystem functioning, plant functional traits, stomatal density, trees

## Abstract

While functional diversity (FD) has been shown to be positively related to a number of ecosystem functions including biomass production, it may have a much less pronounced effect than that of environmental factors or species-specific properties. Leaf and wood traits can be considered particularly relevant to tree growth, as they reflect a trade-off between resources invested into growth and persistence. Our study focussed on the degree to which early forest growth was driven by FD, the environment (11 variables characterizing abiotic habitat conditions), and community-weighted mean (CWM) values of species traits in the context of a large-scale tree diversity experiment (BEF-China). Growth rates of trees with respect to crown diameter were aggregated across 231 plots (hosting between one and 23 tree species) and related to environmental variables, FD, and CWM, the latter two of which were based on 41 plant functional traits. The effects of each of the three predictor groups were analyzed separately by mixed model optimization and jointly by variance partitioning. Numerous single traits predicted plot-level tree growth, both in the models based on CWMs and FD, but none of the environmental variables was able to predict tree growth. In the best models, environment and FD explained only 4 and 31% of variation in crown growth rates, respectively, while CWM trait values explained 42%. In total, the best models accounted for 51% of crown growth. The marginal role of the selected environmental variables was unexpected, given the high topographic heterogeneity and large size of the experiment, as was the significant impact of FD, demonstrating that positive diversity effects already occur during the early stages in tree plantations.

## Introduction

One of the most important aims in functional biodiversity research is to predict the importance of different facets of biodiversity to ecosystem functions (EFs). It has been shown that many different EFs are positively related to producer diversity (Loreau et al. 2001; Hooper et al. 2005; Balvanera et al. 2014). A meta-analysis (Cardinale et al. 2011) revealed that 414 of the 574 independent experimental manipulations of species richness had a positive effect on producer biomass. This also applies to forests, which represent *the* most important ecosystems globally because of their broad geographical cover and the unique ecosystem services they provide (Quijas et al. 2012). A review of worldwide inventories demonstrated positive relationships between forest growth and standing biomass and tree species richness in the majority of published studies (Scherer-Lorenzen 2013). For example, it was shown that biomass production in Swedish forests increases with tree species richness (Gamfeldt et al. 2013).

Such biodiversity-focused research has often tried to minimize environmental variation, which can lead to an underestimation of other major determinants of tree growth such as climate or soil conditions. An analysis of the pan-European tree-ring network showed that forest productivity in central and southern Europe is driven by temperature in high-elevation and high-latitude areas, and moisture at low elevations (Babst et al. 2013). Climate also determines tree growth at the microsite scale with respect to differences in slope, aspect, and inclination (Chen et al. 1999; Geiger et al. 2003). In the Northern Hemisphere, south-facing slopes receive more solar irradiation than north-facing slopes (Warren 2010), resulting in positive effects on individual tree growth (Fralish 1994; Li et al. 2014). As a consequence, each single variable, such as slope aspect, inclination, or altitude, has the potential to significantly affect tree growth (Saremi et al. 2014). In addition, soil conditions have a strong impact on forest productivity (Grier et al. 1989), making soil type a key predictor in forest growth models (Landsberg and Waring 1997; Pinjuv et al. 2006). Such strong dependence of forest productivity on climate and soil conditions indicates that any attempt to detect biodiversity signals on tree growth needs to be separated from effects of the abiotic environmental setting. Accordingly, for the current study, we analyzed a large forest biodiversity experiment on the assumption that the biodiversity treatments only induced broad variation in functional diversities of tree communities across a heterogeneous landscape, and we ignored all other design aspects of the experiment. It is considered that analyzing a designed experiment as if it were a sample survey of plots across the landscape (Snedecor and Cochran 1989) is justified, when the aim is to maximize the environmental impact on ecosystem functioning.

The selected set of environmental variables were related to topography (slope, aspect, and elevation) and soil (pH value, carbon, and nitrogen content in the topsoil), both of which were considered to be relevant to tree growth when the experiment had been established. During the early stage of the experiment, we assumed that the environmental variables had not yet been, or had only been minimally, affected by the experimental biodiversity treatments. This assumption may clearly not apply over a longer term, in particular, given that as microclimate and soil conditions respond to biodiversity in feedback loops, and depending on plot productivity and tree richness, organisms modify their environment (Bruelheide et al. 2014).

Further key determinants of forest production include the particular tree species, as it is well documented that tree growth can vary by an order of magnitude among different tree species (Lieberman et al. 1985; Lambers and Poorter 1992). It is generally assumed that early successional species outperform late successional ones because of higher rates of photosynthesis (Bazzaz 1979). This applies to our study as deciduous species generally grow faster than evergreen ones, and early successional species are often deciduous (e.g., Budowski 1965); however, species growth rates can also differ within successional categories. One approach to better understanding such species identity effects is to relate species-specific differences in growth rates to the species' functional traits (Díaz et al. 2007). It has been shown that a few key traits that describe the leaf economics spectrum (LES) (Wright et al. 2004), such as specific leaf area (SLA) or leaf nitrogen content, can successfully predict tree growth of 53 rainforest species in Bolivia (Poorter and Bongers 2006). At the plot scale, productivity should depend on the mixture of species in the community. According to the mass ratio hypothesis (Grime 1998), the most abundant or dominant species are expected to exert the highest impact on EF. This hypothesis provides the basis for using community-weighted means (CWMs) of trait values, which are obtained from averaging traits at the community level by weighting the species' traits with the species' relative abundance in the given community (Ackerly et al. 2002). In grasslands, this approach has been successfully employed for predicting EF from CWM trait values (Garnier et al. 2004; Roscher et al. 2012). Thus, as one important EF indicator, tree growth may be predicted in relation to the CWM of a single key trait or from a combination of CWMs of different uncorrelated traits.

As CWM represents the overall plot mean, it does not account for trait variation within plots and it fails to capture the effect of functional diversity (FD), both in terms of selection and complementarity effects (Loreau and Hector 2001). In particular, functional diversity may increase resource complementarity and facilitation among species in species-rich plots and thus increase forest productivity (Spasojevic and Suding 2012; Dias et al. 2013). For example, in southern New Zealand, nutrient-rich forest sites were not only characterized by species with high relative growth rates, but also showed higher variation in growth rates related to a high variation in species-specific shade tolerances, resulting in greater complementarity of light use (Coomes et al. 2009). As such, within defined forest age classes, FD has been found to be positively related to aboveground biomass (Bu et al. 2014). Similarly, in the Cedar Creek experiment, functional complementarity of grassland species resulted in higher C and N accumulation in soils (Fornara and Tilman 2007). Complementarity in resource use is expected to emerge in trait space and be reflected by a higher variation and dispersion of values of relevant traits (Lavorel et al. 2008). In principle, a trait can contribute to complementarity of a particular EF in the community, either spatially via above- or belowground resource partitioning (Bessler et al. 2009; von Felten et al. 2009), or temporally via differential resource use in different seasons (Dedeyn and Vanderputten 2005). Trait value distribution in the community can be expressed mathematically by FD measures, such as in the regularity of the distribution of trait abundances (Villéger et al. 2008), as designated by Rao's quadratic entropy (FDQ) (Rao 1982). It should be noted that in communities, the FD of a particular trait is not independent of the CWM of the same trait, as trait variation is constrained by the mean (Dias et al. 2013). In consequence, both the FD and CWM of a single trait explain some degree of variation in EF. Thus, separating FD from CWM poses a similar problem as separating environmental variables from biodiversity effects.

In summary, variation in productivity as an important ecosystem function (EF) in forests may be largely explained by variation in environmental variables, variation in community-weighted mean (CWM) trait values, and variation in functional diversity (FD). The contribution of each of these components on a certain EF can be visualized as a triangle, where environmental variables, CWM, and FD represent the three corners. The location of a particular plant community in this triangular space will depend on the relative impact of the abiotic environment, species-specific properties, and biotic interactions. For example, aboveground net primary production (ANPP) in alpine grasslands was found to be dependent on both nutrient supply (quantified by a nitrogen nutrition index) and FD in vegetative height (which reflects light acquisition complementarity), but not by the CWM of any particular trait (Díaz et al. 2007). Taking all predictors together, 44% of the total variation in ANPP was explained by abiotic conditions alone, and inclusion of FD did not improve the model's explanatory power. However, to our knowledge, no attempt has been made to quantify the contribution of environmental variables, CWM, or FD in forest communities. One important caveat that must be considered in the above context is that it would be unusual for all of the three explanatory corners in the aforementioned triangle to have the same range of variation in any particular study, making it unlikely for any of the factors to have the same chance to influence variation in the dependent variable. In this study, variation was particularly high in CWM and FD because the plots originated from a biodiversity experiment that ensured a large range of species richness levels and, as a consequence, resulted in a large variation in CWM and FD. Nevertheless, the very large topographic and hydrological variation at the experimental site also ensured a high environmental variation.

It might be argued that partitioning the effect of environment, CWM, and FD is only necessary in natural communities but not in designed experiments, where biodiversity is manipulated and environmental variation should be accounted for. To control environmental variation, experimental plots are often established in homogeneous environments, such as a flat piece of land with uniform land-use history and soil properties. However, even comparatively low environmental heterogeneity can strongly affect EF, as was demonstrated in the Sardinilla forest experiment in Panama (Healy et al. 2008). Under environmental heterogeneity, fully randomized experiments cannot prevent certain plots from exhibiting exceptional site conditions. For example, in the Sardinilla experiment, all six-species diversity plots were located at sites with low water drainage (Healy et al. 2008). As homogeneity declines with increasing study size, many experiments have employed blocking techniques, for example, with respect to distance from the river in the Jena Experiment (Roscher et al. 2004). However, blocking is only useful when there are few and clear gradients across the experimental site (Bruelheide et al. 2014), and in very heterogeneous environments, blocking may not be feasible. In many regions of the world, forests mainly occur in topographically heterogeneous environments, as the often more fertile flat lands are used for agriculture (Sandel and Svenning 2013). As such, in field-based forest biodiversity–EF experiments, such environmental effects are often confounded with biodiversity and have to be accounted for in the same way as in natural communities.

The aim of our study was to partition the effects of 11 environmental variables and CWM and FD variables calculated from 41 species traits to one key ecosystem function in the early stage of a large forest-based biodiversity experiment in subtropical China (Bruelheide et al. 2014). A set of 40 broadleaved tree species native to the natural vegetation was planted in richness levels of 1, 2, 4, 8, 16, and 24 tree species. We used results from one of two sites, which had been planted in 2009 with 1-year-old saplings (Yang et al. 2013; Bruelheide et al. 2014). To measure tree productivity, we chose mean annual crown width growth between 2011 and 2012, as it best reflected tree growth at the early growth stage of the experiment (Li et al. 2014). We expected forest growth at this early stage to be mainly dominated by abiotic conditions, based on the finding of Li et al. (2014) that growth of individual trees is related to aspect and soil nitrogen content but not to Shannon diversity of the local tree neighborhood. Crown width growth rate data referred to Li et al. (2014), and data were aggregated at the plot level, with plot mean values being subjected to the analysis framework of Díaz et al. (2007). A stepwise approach was used to disentangle the effects of the three groups of predictor variables mentioned above, as suggested by Díaz et al. (2007) and by sequentially fitting the influence of different predictors of the environment, CWM, and FD. The objective of our study was to identify the single environmental, CWM, and FD predictors that best predicted plot-level tree growth. In particular, we hypothesized 1) that there are single variables from the three predictor groups (environmental variables, CWM, and FD) that significantly explain tree growth and 2) that, comparing the best predictors from the three groups, the environmental variables have the highest explanatory power for tree growth rate at the early stage of the experiment. To our knowledge, our study is the first to disentangle the effect of environmental variables, CWM, and FD in a biodiversity functioning experiment with trees. Our results are the first ones on trait–EF relationships from all forest diversity experiments worldwide.

## Materials and Methods

### Study site

We conducted our study on a field experiment (BEF-China) in southeast subtropical China (29.08–29.11 N, 117.90–117.93 E). BEF-China is a large-scale biodiversity and ecosystem functioning study on subtropical tree species (Yang et al. 2013; Bruelheide et al. 2014), which was established on the site of former *Pinus massoniana* and *Cunninghamia lanceolata* conifer plantations that were harvested at ∼20-year intervals. After clear-cutting the conifer plantations, aboveground plant biomass was removed from the study site (Yang et al. 2013) and a pool of 40 species native to the regional broadleaved forest was established across 38 ha in 2008/2009. The diversity gradient employed comprises monocultures and plots with 2, 4, 8, 16, and 24 species. Here we present the results of one of the two experimental sites (Site A), for which we evaluated data on tree growth measurements from 231 plots (25.8 × 25.8 m) and 23 species planted at the site. We analyzed annual increment of crown diameter as the response variable, which was calculated from two monitoring sessions undertaken in 2011 and 2012 (Li et al. 2014). We used data on 23 species, of which 14 and 9 species were deciduous and evergreen, respectively. Accordingly, the majority of species was classified as early successional (Li et al. 2014), and none of the species were N_2_-fixing. All growth data were aggregated at the plot level by taking the arithmetic mean of the absolute crown diameter increment across all individuals measured in each plot.

### Assessment of environmental variables

A 5 m digital elevation model (DEM) was established based on differential GPS measurements carried out in 2009. The DEM was used to derive plot mean values for elevation, aspect, mean slope, solar insolation, profile curvature, and plan curvature as descriptions of the environmental conditions (Evans 1979; Zevenbergen and Thorne 1987; Dietrich and Montgomery 1998; Shary et al. 2002). Sine and cosine transformations of the aspect were used to express eastness and northness, respectively (Roberts 1986). All calculations were made using ArcGIS 9.0 (ESRI Corp., Redlands, CA).

Soil variables were based on nine soil samples per plot, collected in 2010 by taking soil cores at a depth of 0–5 cm. The nine soil samples per plot were thoroughly mixed, and one bulk sample per plot was analyzed for total carbon (C) and total nitrogen (N) content. Prior to the chemical analysis, soil samples were air-dried and sieved (<2 mm). For the C and N analyses, dry soil samples were ground with a ball mill and subjected to total C/N analysis based on gas chromatography (Vario EL, Elementar, Hanau, Germany). Minimum, maximum, and mean values and standard deviation of all environmental variables is shown in Table S1.

### Assessment of leaf traits

All traits were used to calculate community-weighted means (CWMs) and functional diversity (FD) as predictive variables. These included the following: (1) traits connected to the leaf economics spectrum, such as specific leaf area (SLA) and leaf nitrogen content (LNC); (2) traits related to stomatal conductance, such as maximum and mean stomatal conductance; (3) traits related to xylem properties, such as specific hydraulic conductivity of the xylem (K_S_) and the xylem pressure at which 50% loss of the maximum specific hydraulic conductivity occurred (Ψ_50_); and (4) leaf microscopic traits, such as stomata density and thickness of the palisade parenchyma. In assessing leaf traits, only sun-exposed, fully developed, and nondamaged leaves were sampled, with at least five individuals per species being sampled. Traits related to stomatal conductance were extracted from diurnal measurements on tree individuals from the same experiment. Traits directly related to stomatal conductance, such as stomata density and size, were assessed on the same leaves from which measurements of stomatal conductance were taken. Three individuals per species were sampled to generate the xylem dataset. The wood traits were measured on the same wood samples that were assessed for xylem hydraulic measurements. The complete trait datasets and the specific measurement protocols were provided by Kröber and Bruelheide (2014); Kröber et al. (2014a,b).

### Statistics

To test for spatial autocorrelation between the plots, Moran's I was calculated, using the ape package in R (http://cran.r-project.org/web/packages/ape/index.html). To test for interrelationships between all traits, we calculated Gower's distance between traits, which allows for the processing of traits of different scales using the ade4 package of R (Dray & Dufour 2007). Thereafter, a principal coordinate analysis (PCoA) was carried out, and the correlations between traits and PCoA axes were obtained by post hoc correlation using the envfit function in the vegan package (Oksanen et al. 2013).

CWM values of traits were calculated according to Garnier et al. (2004) and FD_Q_ (Rao's quadratic entropy) according to Botta Dukát (2005). Both CWM and FD_Q_ were weighted by the frequency of the tree species in each plot. According to the design of the BEF-China experiment, all tree species in each plot were represented by the same number of trees. However, due to mortality, the number of trees per species on which growth rates were measured varied somewhat, so we made sure that the same proportions of trees that were used to calculate plot means of crown diameter growth rates were also used for calculating CWM and FD. All predictor variables were scaled by mean and standard deviation, which allowed for the interpretation of the effect sizes with regard to their importance on crown width growth rate. In a first step, we analyzed the impact of each single predictor on crown diameter increment using separate linear models. We then tested the trait complexes in combination according to Díaz et al. (2007) for their explanatory power in predicting crown growth. As many different trait combinations can equally explain plant growth (Marks and Lechowicz 2006a,b), we tested all possible combinations of the predictor variables and then selected the best model that had a maximum of five predictor variables based on Akaike's information criterion, corrected for small sample sizes (cAIC) using the MuMIn package in R (Barton 2014). The independent effect of each predictor variable in the final model on crown growth was assessed by plotting the residuals of crown width growth rates against this predictor variable. The residuals were obtained from a model that contained all predictors except the focus variable. Finally, to test the impact of the three predictor groups (environment, CWM and FD), we applied variance partitioning with all the significant predictors using the vegan package in R (Oksanen et al. 2013). For all statistical analyses, we used the software R version 3.1.0 (R Core Team 2014).

## Results

**Figure 1:**
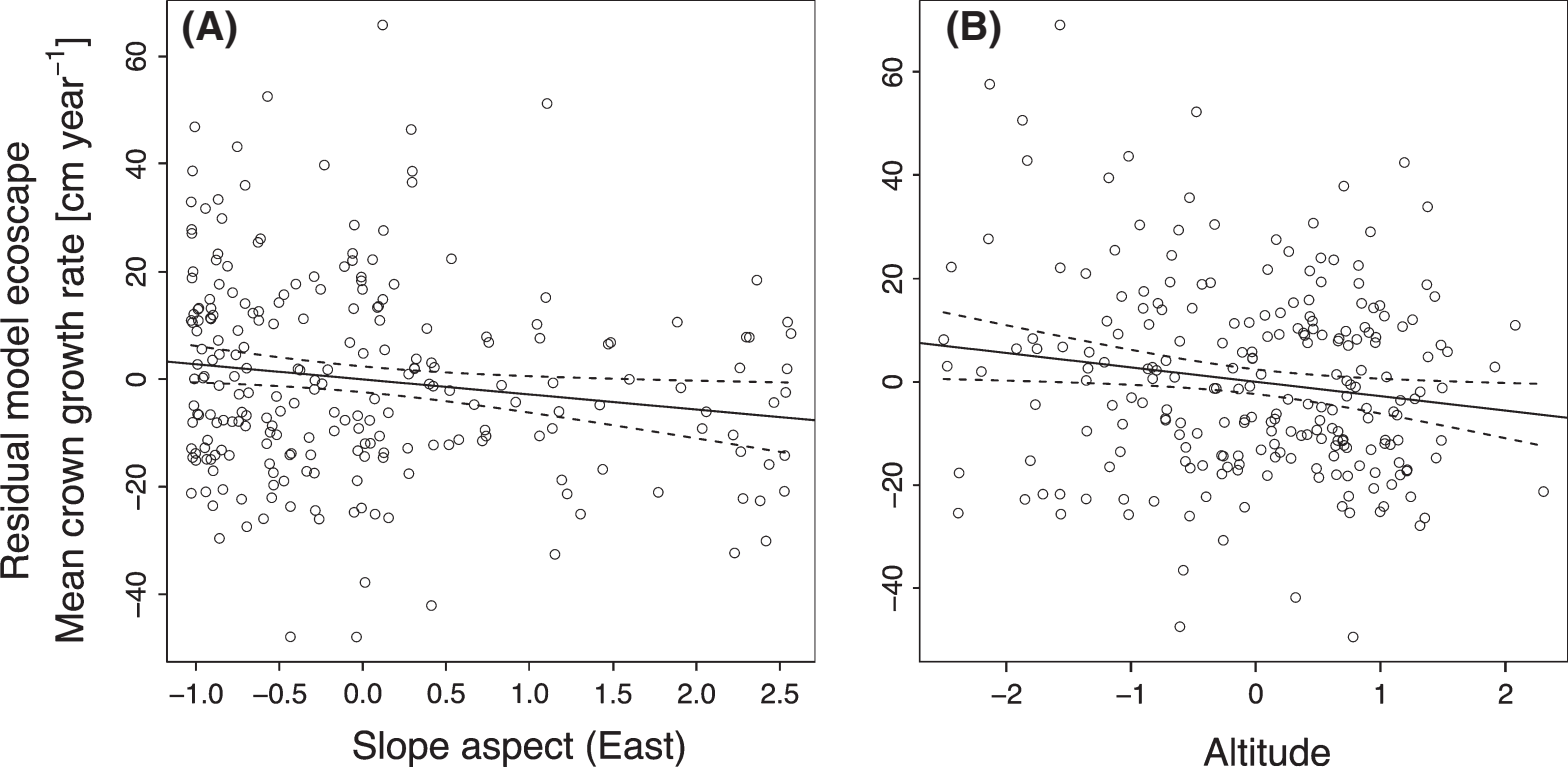
Mean annual crown width growth rate as predicted by the environment multipredictor model. The residuals from all other terms in the model are plotted against (A) slope aspect (East) and (B) altitude. Each dot represents a single plot. All predictor variables are scaled by mean and standard deviation; as such, the slope of the regression shows the direction and magnitude of impact on CW growth rates. The panels have been arranged in the sequence of decreasing order of effect sizes. For statistical details, see Table3.

The analysis of spatial autocorrelation of tree crown diameter increment showed a Moran's I value of 0.0059, which was not significantly different (*P* = 0.1128) from the expected value (−0.0044). Contrary to our expectations, environmental factors had no significant effects on plot means of annual crown width growth rates (Table1). The best environmental predictor was slope inclination (SLOPE), which nonetheless only had a marginally significant effect on crown width (CW) growth rates (*P* = 0.091). Testing for combinations of all environmental variables in all possible multipredictor models, the minimal model only retained altitude and eastness, both of which had a negative impact on tree growth (Table 3). This means that the plot mean of tree crown diameter growth at the plot level was larger at low elevations (valleys and foot slopes) and on west-facing slopes than high elevations and east-facing slopes (Fig.1). However, the minimal environmental model only explained 3.8% of the total variation in crown width growth rates (Table 3).

**Table 1:** Impact of environmental variables on crown growth

Abbreviation	Predictor	Source	Estimate	*r* ^2^	*P*
ALT	Altitude	DEM	−0.05	0.01	0.11
SLO	Slope	DEM	−0.38	0.01	0.09
SOLAR	Solar radiation	DEM	0.00	0.00	0.31
CURV X	Profile curvature	DEM	0.02	0.00	0.50
CURV Y	Plan curvature	DEM	0.00	0.00	0.94
NORTH	Aspect northness	DEM, cosine of slope	−0.47	0.00	0.81
EAST	Aspect eastness	DEM, sine of slope	−3.62	0.01	0.11
PH	Soil pH (KCl)	Soil sampling, pH electrode	0.80	0.00	0.92
N	Soil nitrogen content	Soil sampling, total CN analyzer	13.99	0.00	0.71
C	Soil carbon content	Soil sampling, total CN analyzer	−0.28	0.00	0.88
CN	Soil carbon nitrogen ratio	Soil sampling, total CN analyzer	−0.31	0.00	0.62

The effect of environmental predictors on crown width growth rate, assessed as plot mean values between 2011 and 2010. All environmental variables are scaled by mean and standard deviation; as such, the estimates show the direction and magnitude of impact on CW growth rates. DEM: digital elevation model.

**Figure 2:**
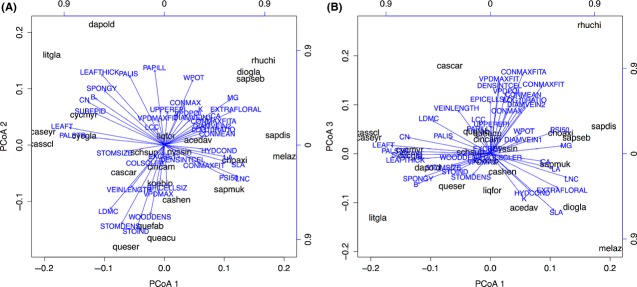
Principal coordinate analysis (PCoA) biplots of the traits listed in Table2. (A) PCoA axes 1 and 2, and (B) PCoA axes 1 and 3. See Table2 for coding of trait names. Eigenvalues: axis 1 = 0.352, axis 2 = 0.236, axis 3 = 0.208, with cumulative proportion of explained variance 20.9, 34.9, and 47.2%, respectively. Species abbreviations refer to genus and species epithet; see supplementary material Table S2 for full species names.

**Figure 3:**
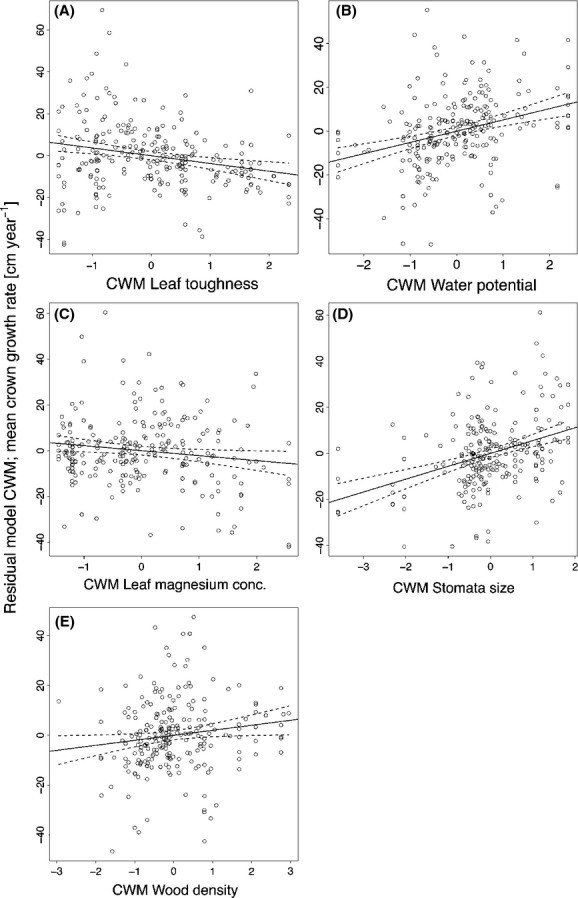
Mean annual crown width growth rate as predicted by the CWM multipredictor model. The residuals from all other terms in the model are plotted against (A) leaf toughness, (B) water potential, (C) leaf magnesium content, (D) stomata size, and (E) wood density. Every dot represents one plot. All predictor variables are scaled by mean and standard deviation; as such, the slope of the regression shows the direction and magnitude of impact on CW growth rates. The panels have been arranged in the sequence of decreasing order of effect sizes. For statistical details, see Table3.

The results of the principal coordinate analysis (PCoA) of all 41 traits are shown in Figure2A,B for axis one versus two and one versus three, respectively. The first axis reflected the leaf economics spectrum with leaf toughness and C/N ratio versus SLA, while the second axis was characterized by wood density and LDMC and the third axis by leaf hydraulics. Numerous traits had significant effects on plot tree growth, which was also evident by the inclusion of many CWM and FD predictors in the minimal multiple regression models (Table 3). In total, 25 and 15 of the 41 variables produced significant single predictor models for CMW and FD, respectively (Table2). The best single CWM predictor for CW growth rates was number of palisade parenchyma layers (PALSTR, *r*^2^ = 0.24), while the best FD predictor was the presence of extra-floral nectaries (EXTRAFLORAL, *r*^2^ = 0.10), a trait only encountered in four of the 23 species (i.e., *Diospyros japonica, Melia azedarach, Triadica cochinchinensis,* and *T. sebifera*, Table S2). Many significant CWM predictors were typical traits of the LES, such as specific leaf area (SLA), leaf nitrogen, potassium and magnesium content (LNC, K, MG), and the leaf carbon to nitrogen ratio (CN). However, except for magnesium, these variables had lower estimates compared to morphological and anatomical variables such as leaf toughness, leaf dry matter content (LDMC), leaf thickness, the presence of a subepidermis, number of palisade parenchyma layers, and the presence of a column of sclerenchyma cells through the leaf (Table2). In the minimal multipredictor model (Table3), some of these variables, such as water potential (WPOT), stomata size (STOMSIZE), or wood density (WOODDENS), had positive effects on crown width growth rates, while leaf toughness (LEAFT) and leaf magnesium content (MG) had negative effects (Fig.3). A principal component analysis revealed that trait interrelationships did not influence the final minimal model (see PCA scores in Table S3A, S3B, S3C).

**Table 2:** Impact of CWM and FD on crown growth

			CWM			FD		
Abbreviation	Predictor	Source	Estimate	*r* ^2^	*P*	Estimate	*r* ^2^	*P*
PSI50	Loss of 50% initial flowrate	Scholander pressure bomb	0.84	0.00	0.50	−0.40	0.00	0.75
K_S_	Maximum flowrate	Laboratory measurements	2.46	0.02	0.05	5.19	0.08	0.00
B	Parameter b (Sigmoid Regression)	Scholander pressure bomb	−6.20	0.11	0.00	−3.44	0.03	0.01
CONMEAN	Average stomatal conductance	Steady state porometer	5.27	0.08	0.00	2.66	0.02	0.03
CONMAX	Maximum stomatal conductance	Steady state porometer	3.99	0.05	0.00	1.21	0.00	0.33
VPDMAX	Vpd at CONMAX	Steady state porometer	−0.82	0.44	0.51	−0.32	0.00	0.80
CONMAXFIT	Relative fitted Max. stomatal conductance	Steady state porometer	2.32	3.57	0.06	1.62	0.01	0.19
CONMAXFITA	Absolute fitted Max. stomatal conductance	Steady state porometer	5.00	0.07	0.00	2.73	0.02	0.03
VPDMAXFIT	Vpd at CONMAXFIT	Steady state porometer	1.73	0.01	0.16	0.25	0.00	0.84
VPDPOI	Vpd at point of inflection of fitted stomatal conductance	Steady state porometer	0.50	0.00	0.69	0.29	0.00	0.81
WOODDENS	Wood density	Laboratory measurements	0.42	0.00	0.74	4.42	0.06	0.00
WPOT	Water potential	Scholander pressure bomb	7.74	0.17	0.00	4.68	0.06	0.00
LA	Leaf area	Laboratory measurements	1.10	0.00	0.38	4.55	0.06	0.00
LDMC	Leaf dry matter content	Laboratory measurements	−7.24	0.15	0.00	2.09	0.01	0.09
SLA	Specific leaf area	Laboratory measurements	4.05	0.05	0.00	0.66	0.00	0.59
LEAFT	Leaf toughness	Leaf toughness device	−7.65	0.17	0.00	−0.40	0.00	0.75
STOMDENS	Stomata density	Microscope	−3.40	0.03	0.01	1.80	0.01	0.15
STOMSIZE	Stomata size, ellipse from stomata length and width	Microscope	1.87	0.01	0.13	1.24	0.00	0.31
STOIND	Stomata index	Microscope	−2.95	0.02	0.02	1.82	0.01	0.14
LNC	Leaf nitrogen content	CN analyzer	2.88	0.02	0.02	−1.99	0.01	0.11
LCC	Leaf carbon content	CN analyzer	0.62	0.00	0.62	0.73	0.00	0.56
CN	Leaf carbon nitrogen ratio	CN analyzer	−3.48	0.03	0.00	−0.80	0.00	0.52
CA	Leaf calcium content	Atom absorption spectrometer	−1.55	0.01	0.21	3.35	0.03	0.01
K	Leaf potassium content	Atom absorption spectrometer	4.30	0.05	0.00	−0.19	0.00	0.88
MG	Leaf magnesium content	Atom absorption spectrometer	6.78	0.13	0.00	1.27	0.00	0.31
DIAMVEIN1	Diameter veins 1st order	Microscope	2.41	0.02	0.05	0.79	0.00	0.53
DIAMVEIN2	Diameter veins 2nd order	Microscope	3.88	0.04	0.00	2.75	0.02	0.03
VEINLENGTH	Length of first-order veins per cm^2^	Microscope	−3.09	0.03	0.01	4.49	0.06	0.00
UPPEREPI	Upper epidermis thickness	Microscope	−1.28	0.00	0.30	0.78	0.00	0.53
PALIS	Palisade parenchyma thickness	Microscope	−3.73	0.04	0.00	2.70	0.02	0.03
SPONGY	Spongy parenchyma thickness	Microscope	−3.88	0.04	0.00	1.42	0.01	0.25
LOG10RATIO	Log ratio of the palisade to spongy parenchyma thickness	Microscope	0.28	0.00	0.82	2.81	0.02	0.02
LEAFTHICK	Leaf thickness	Microscope	−5.33	0.08	0.00	1.57	0.01	0.21
SUBEPID	Presence of a subepidermis	Microscope	−5.32	0.08	0.00	−3.96	0.04	0.00
EPICELLSIZ	Ratio of the cell size of upper and lower epidermis	Microscope	4.58	0.06	0.00	−2.37	0.02	0.05
PALSTR	Number of palisade parenchyma layers	Microscope	−9.10	0.24	0.00	−1.01	0.00	0.41
EXCRET	Presence of excretory glands	Electron microscope	−0.11	0.00	0.93	0.05	0.00	0.97
DENSINTCEL	Density of spongy parenchyma	Microscope	1.33	0.01	0.28	2.35	0.02	0.06
COLSCLER	Presence of a column of sclerenchyma cells through the leaf	Microscope	−5.30	0.08	0.00	−3.93	0.04	0.00
PAPILL	Presence of papillae	Electron microscope	−3.06	0.03	0.01	0.36	0.00	0.77
EXTRAFLORAL	Presence of extra-floral nectaries	Observation	1.85	0.01	0.13	5.96	0.10	0.00

The effect of community-weighted mean (CWM) values and functional diversity (FD) of single traits on crown width (CW) growth rate, assessed as plot mean values between individual differences crown width in 2011 and 2010. All variables are scaled by mean and standard deviation; as such, the estimates show the direction and magnitude of impact on CW growth rates.

**Table 3:** Multipredictor model coefficients for environmental variables, CWM, and FD

Model	*r* ^2^	Significant predictors	Abbreviation	Estimate	*P*
Environment	0.04	Altitude +	ALT	−0.08	0.0129
		Aspect (east)	EAST	−6.03	0.0125
CWM	0.44	Leaf toughness +	LEAFT	−14.5	<0.001
		Leaf magnesium content +	MG	−11.1	<0.001
		Stomata size +	STOMSIZE	7.2	<0.001
		Wood density +	WOODDENS	2.9	0.0103
		Water potential	WPOT	13.0	<0.001
FD	0.31	Extra-floral nectaries +	EXTRAFLORAL	3.93	0.0011
		Number of palisade layers +	PALSTR	−8.41	<0.001
		Stomata index +	STOIND	−11.46	<0.001
		Vein length +	VEINLENGTH	9.27	<0.001
		Water potential	WPOT	12.14	<0.001
Combined	0.51	Altitude + aspect (east) + leaf toughness + leaf magnesium content + stomata size + wood density + water potential + extra-floral nectaries + number of palisade layers + stomata index + vein length + water potential			

Results of the minimum multipredictor models for environmental variables, community-weighted mean (CWM) values, and functional diversity (FD) and the overall model combining these three multipredictor models. All variables are scaled by mean and standard deviation; as such, the estimates show the direction and magnitude of impact on CW growth rates.

Significant FD variables were essentially a subset of the significant CWM variables, except for wood density, leaf area (LA), leaf calcium content (CA), the ratio of palisade to mesophyll layer thickness (LOG10RATIO), and the presence of extra-floral nectaries (EXTRAFLORAL), for which only FD but not CWM had a significant effect on CW growth rate. In addition, there were two variables, hydraulic conductance (K_S_) and vein length (VEINLENGTH), for which FD had a higher explanatory power than CWM.

**Figure 4:**
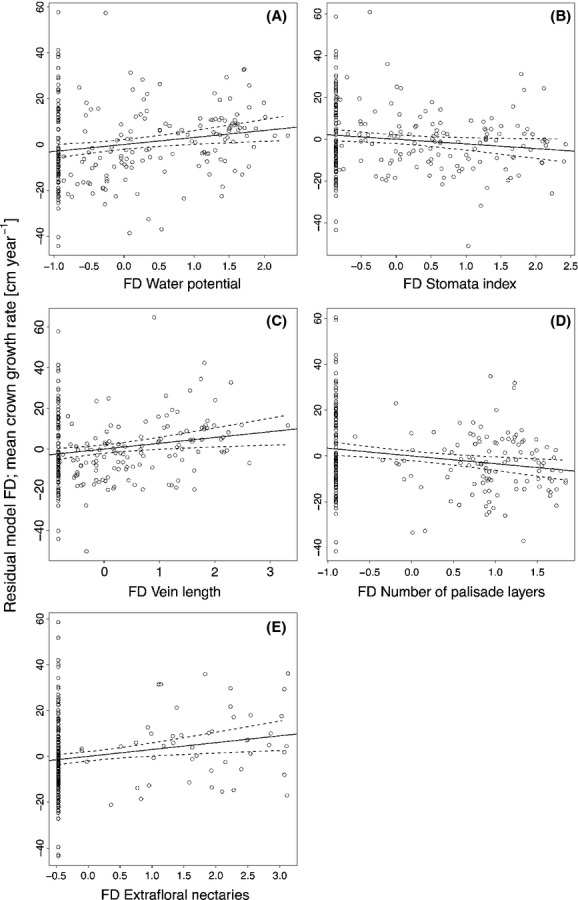
Mean annual crown width growth rate as predicted by the FD multipredictor model. The residuals from all other terms in the model are plotted against (A) water potential, (B) stomata index, (C) leaf vein length, (D) number of palisade parenchyma layers, and (E) the presence of extra-floral nectaries. Every dot represents one plot. All predictor variables are scaled by mean and standard deviation; as such, the slope of the regression shows the direction and magnitude of impact on CW growth rates. The panels have been arranged in the sequence of decreasing order of effect sizes. For statistical details, see Table3.

Interestingly, the minimal multipredictor model for the FD–growth relationship included variables with both positive (WPOT, VEINLENGTH, EXTRAFLORAL) and negative effect sizes, such as stomata index (STOIND) and number of palisade layers (PALSTR, Fig.4).

**Figure 5:**
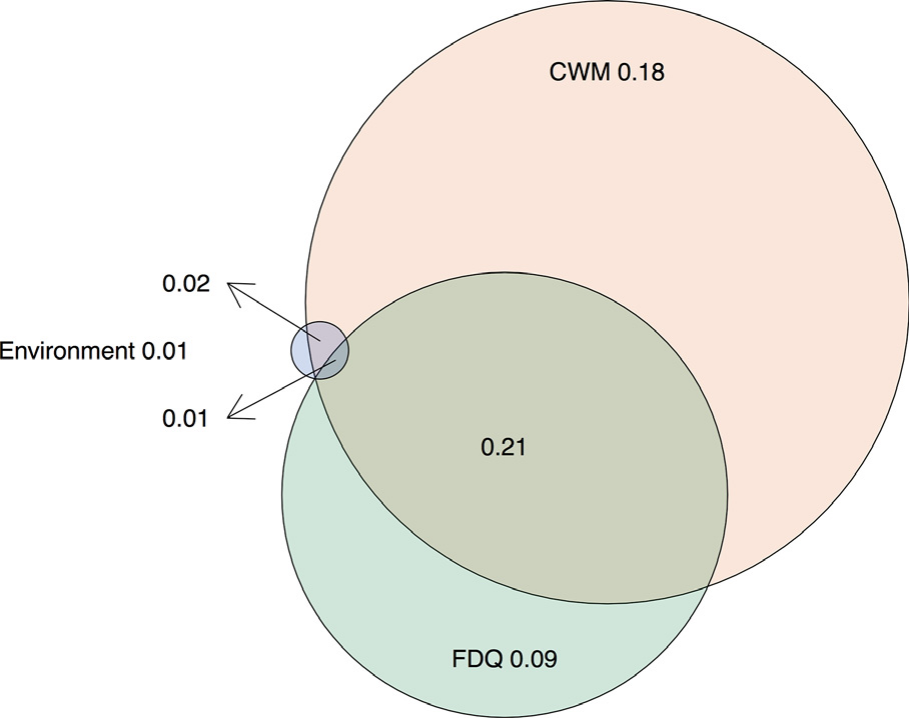
Plot of the partitioned variance explained by the three different variable complexes, green = environment, purple = CWM, light blue = FD; values below 0.01 not shown. For statistical details, see Table3.

In combination, the three best multipredictor models of environment, CWM, and FD explained 51% of variation of plot-level crown width growth rates (Fig.5). CWM explained most variation, both in terms of exclusive impact on tree growth that was not captured by environment or FD and in terms of shared variance with environment and FD. For example, of the 31% variance in crown diameter growth explained by FD, two thirds (i.e., 22%) were also shared by CWM predictors. There was almost no variation left that was exclusively explained by environment (1%).

## Discussion

Using the combined information of selected environmental variables, community-weighted means, and functional diversity, we could account for 51% variability of crown width growth rates. Contrary to expectations, no single variable explained crown diameter growth to a sufficient degree. The largest proportion of variance explained by a single variable was 24% (number of palisade parenchyma layers). Nevertheless, our first hypothesis was confirmed as we identified some single key variables for tree growth, albeit with the most powerful ones being based on community mean trait values. In contrast, environmental variables turned out to be weak predictors for crown growth and explained less than 4%, while functional diversity explained up to 31% and community-weighted means up to 42% of crown growth. Thus, we have to reject our second hypothesis of a dominant impact of environment on tree growth at this early stage of the experiment.

The low importance of environment was unexpected, given the high topographic heterogeneity and large size of the experiment. Altitude had a negative impact on tree growth, which was opposite to the findings on initial tree survival at the same site (Yang et al. 2013). Altitude affected productivity in a similar way in the Sardinilla study from central Panama (Healy et al. 2008). In Sardinilla, the single environmental variable with the highest impact on productivity was slope inclination, followed by water drainage quality. Total variance of productivity explained by environment in Sardinilla was 35%. However, the Sardinilla plots only differed by 8 m in altitude, while our site varied by 170 m, but with a total experiment size of 26.7 vs. 8 ha in Sardinilla (Bruelheide et al. 2014). Interestingly, many environmental variables with reported effects in the literature did not have any significant impact on plot mean crown diameter growth in the BEF-China experiment, such as pH. Soil pH is well-known to limit nutrient availability (Lambers et al. 2008) and was found to limit tree growth in primeval forests in the Changbaishan in northeastern China (Yang et al. 2009). Increasing soil pH, in addition to increasing elevation, showed negative effects on aboveground biomass increment in tropical Andean forests (Unger et al. 2012).

The negative effect of higher elevation might be explained by a temperature gradient, with lower temperatures at higher elevations being disadvantageous in winter and spring, or lower slope locations being more sheltered from wind. However, there is no indication that elevation has indirect effects via differing soil conditions, as all these did not result in significant models. The fact that higher crown width growth rates were observed in plots on west-facing slopes may be due to the longer lasting effects of morning dew in summer, which might result in lower values of vapor pressure deficit (vpd) in the morning, which in turn would allow trees to have a prolonged period of gas exchange and, consequently, higher rates of carbon assimilation before stomata closure occurs at increasing vpd values. We have to consider that the selection of environmental variables included in this study might not have captured the key environmental drivers for tree growth. For example, direct microclimate measurements would have been desirable; however, we expect that microclimate reflects topography and would show strong differences between north- and south-facing slopes. Similarly, further nutrients would be expected to covary with total soil C concentration, which had no effect on tree growth.

With respect to community-weighted means (CWMs), we found traits of the leaf economics spectrum (LES) to affect tree growth, with a positive effect shown for specific leaf area (SLA) and leaf magnesium content (MG). Nonetheless, morphological and anatomical traits, such as leaf toughness and thickness, number of palisade parenchyma layers, and the presence of a subepidermal layer, had a higher explanatory power than typical LES traits. The number of palisade parenchyma layers had already been identified as a good proxy for maximum stomatal conductance (CONMAX) (Kröber et al. 2014a), and thus, increasing tree growth would have been expected with increasing number of palisade parenchyma layers. The best 5-predictor model for CWM variables comprised two traits of leaf morphology (leaf toughness and stomata size) and one of plant hydraulics (water potential), while only Mg concentration (MG) was included as a typical LES trait and wood density as a key trait of the wood economics spectrum (Baraloto et al. 2010; Freschet et al. 2010; Martínez-Cabrera et al. 2011). This complex of morphological, anatomical, and physiological traits supports the idea that integrating more and novel functional traits might increase the predictability of ecosystem functioning and, consequently, the reliability of products based on these relationships, such as dynamic vegetation models (Scheiter et al. 2013). As the different variables in the multipredictor model explained additional variation in crown width growth rates, they were not fully collinear to each other, showing that the leaf and wood economics spectrum did not perfectly match (Baraloto et al. 2010). The comparably low explanatory power of LES traits on tree growth in the single predictor models and their contrasting role in the multipredictor model challenges the assumption of a universal positive growth–LES effect on tree growth. Trees may also behave differently from herbaceous plants, particularly where strong positive growth–LES relationships have been described (Grime and Hunt 1975; Poorter and van der Werf 1998). The low predictive power for tree growth has been recently demonstrated in a meta-analysis that estimated size-standardized relative growth rates for 278 tree species from 27 sites around the world and found no significant relationship to SLA or wood density (Paine et al. 2015). Another variable in the best multipredictor model was stomata size, which enables species to attain maximum stomatal conductance at low vpd values (Kröber and Bruelheide 2014). Furthermore, tree crown growth was positively related to xylem water potential measured in the field, showing that species grew more vigorously when they were able to keep their water status at a more moderate level. This was also reflected in leaf toughness, which had the highest explanatory power in the multipredictor model and can be interpreted as a key defense trait against herbivores (Kursar and Coley 2003). In our model, species grew better when they invested less in physical defense.

Although we encountered approximately three times the amount of significant relationships between CWM than FD to single traits, there were several FD traits that showed significant effects on crown diameter increment that complimented CWM effects. The significance of FD at this early stage shows that effects of complementarity in resource use have already emerged. In principle, the traits with significant FD effects on tree growth can be thought to operate through spatial complementarity, such as wood density and leaf area. The joint occurrence of species with low and high investment in wood allows a community to quickly build up tall canopies with fast-growing species while forming a second layer of more slowly growing, durable-wood species. In Iberian forests, canopy trees with denser wood had lower maximum height and wider crown widths (Poorter et al. 2012b). As wood density and physiological strategies of trees are closely related (Santiago et al. 2004), a wide range of wood density in a plot might increase the total amount of resources captured in this plot. Leaf area plays a central role in leaf trait relationships because the mass-normalized traits in the leaf economics spectrum are proportional to leaf area (Osnas et al. 2013). Thus, leaf area might represent a sum variable that captures variance in other variables in the LES as well as morphological traits, such as palisade parenchyma thickness (PALIS) and the palisade to mesophyll ratio (LOG10RATIO). Particularly in young plantations, large-leaved species can quickly increase a stand's leaf area index, while species with smaller leaves follow a more invariable investment strategy. Studies on crown filling in the BEF-China experiment are still ongoing, but results from natural forests revealed that diverse plots have a higher crown overlap than species-poor plots (Lang et al. 2012). Species with small leaves also tend to be evergreen (Kröber et al. 2014a) and may also be complementary to large-leaved deciduous species in time. In our study, traits that potentially contribute to temporal complementarity were all related to plant water relations, such as specific hydraulic conductance of the xylem (K_S_), xylem water potential, leaf vein length and diameter, and leaf stomatal conductance. FD in these water flux-related traits can increase growth rates where some species display high carbon assimilation rates under optimal humid conditions, while others continue with carbon sequestration in dry spells, which frequently occur in summer at the experimental site (Zhou et al. 2011, 2013). Interestingly, FD of some traits also had negative effects on crown width growth rates, such as slope of the xylem vulnerability curve (B), the presence of a subepidermis, and the presence of columns of sclerenchyma cells in the leaf. In principle, negative estimates of FD can only be interpreted as corroborating the CWM signal of these traits, which was also negative in all these cases. While positive FD effects can only arise when growth is higher in mixtures than in monocultures, irrespective of the traits considered, negative FD effects only occur when the traits considered promote growth toward the extreme values where CWM and FD show a strong covariation (Dias et al. 2013). In addition, some traits may act through facilitation, that is, by enhancing the growth of different species' individuals. Such a trait is most likely to be the presence of extra-floral nectaries, which was the FD trait in the single predictor models with the highest impact on crown width growth. Interestingly, the presence of one of the four species with such nectaries in a plot increased overall plot mean crown growth rates. Extra-floral nectaries have previously been shown to have large effects on plant performance through ant–plant mutualism, as ants attracted by extra-floral nectaries have been found to reduce infestation levels of herbivores (Oliveira 1997). While the presence of extra-floral nectaries be beneficial for the target plant itself (Kersch-Becker et al. 2013; Pereira and Trigo 2013), they may have positive effects at the community level (Koptur 1992). The multipredictor model for the FD–growth relationship included the presence of extra-floral nectaries, which can be interpreted as facilitation. Further variables included in the model point to temporal complementarity, while most variables related to spatial complementarity did not enter the final model.

Combining environment, CWM, and FD in the overall model confirms that environment has a very minor bearing on tree growth, which contrasts with Li et al. (2014), who carried out single-tree-level analyses at the same site and using the same crown width growth data as that for our study. This indicates that individual trees respond much more strongly to topography and soil than plot-level mean growth. However, the overall contribution of soil variables, such as C and N content, on individual tree growth was also low at the single tree level (Y. Li, unpubl. data). A further reason for the discrepancy between plot and single-tree-level data was that Li et al. (2014) treated species as a random factor and consequently assigned all trait differences between species to random variation, whereas we accounted for such differences in CWM functional traits. Similarly, FD was also only partially represented in the single-tree models of Li et al. (2014) by including Shannon diversity of the local neighborhood, which was found to not contribute to explaining crown growth. Thus, FD may capture more unexplained variation than Shannon diversity. In addition, FD effects may only play out when scales larger than the immediate neighborhood are considered, that is, on whole plots that contained 400 tree individuals.

It should also be noted that the environmental variables included in our study did not sufficiently reflect resource supply. While many variables showed only a very low amount of variation among plots, such as soil pH, other soil variables such as nitrogen content or carbon to nitrogen ratio might reflect the preplanting conditions of the conifer plantations of *Pinus massoniana* and *Cunninghamia lanceolata*, which may have levelled out differences among plots. As such, some key variables for tree growth such as phosphorus supply have been excluded in our study. However, considering that soil variables are known for their strong spatial autocorrelation (Hengl et al. 2004), we would not expect them to have such a large impact on crown diameter increment, as this response variable was randomly distributed in space.

Although our overall model explained 51% of variation in plot-level crown growth, a substantial amount of variation in the growth–trait relationship remained unexplained, which is typical of tree growth studies (ter Steege 2003; Poorter et al. 2008; Martínez-Vilalta et al. 2010; Wright et al. 2010; Rüger et al. 2012). Possible causes for unexplained variation might include negative biotic interactions such as pathogens or herbivores, both of which reduce potential growth rates. As such, field studies most probably produce different conclusions to that of greenhouse trials, which assess potential growth rates under the exclusion of biotic interactions and show strong relationships to functional leaf traits (Böhnke and Bruelheide 2013). Moreover, juvenile trees may allocate resources to the expansion of their root system for several years without showing any substantial aboveground growth, especially in dry or nutrient-poor forests (Poorter et al. 2012a).

## Conclusion

It is important to note that our study was conducted at an early stage of the experiment, at which time the system is neither stable nor in equilibrium, and as trees continue to grow, tree–tree interactions will become increasingly intense. At present, forest growth is still dominated by CWM effects, but an increasing impact of FD at the expense of CWM effects may be expected in the future. The role of the environment is, however, unpredictable. A distinction can be made between environmental variables that are temporally invariable (such as slope, aspect, elevation) and those that are dynamic (such as microclimate, content of soil organic matter and nutrients, and soil reaction). While invariable abiotic site conditions are not important at present, dynamic environmental variables will become increasingly affected by tree growth. Thus, we expect that tree growth feeds back on this aspect of the environment. In particular, with respect to biodiversity, the dynamic environment can take the form of a positive feedback loop, whereby a higher variation of organisms that depend on higher tree richness modifies the abiotic environment to their own favor. Therefore, it may be the case that diversity creates conditions that are amenable to more diversity. In this respect, the process might be similar to niche construction models for single species discussed by Odling-Smee et al. (2003). Where biodiversity has an effect by modifying the environment, an increase in the importance of the dynamic environment components can be expected.

## Supporting Information

Table S1. Plot data. **Table S2.** Tree species data.

Table S3. Principal Component Scores.
