# Supplementary material for: Early subtropical forest growth is driven by community mean trait values and functional diversity rather than the abiotic environment
Source: Ecol Evol. 2015 Aug 6;5(17):3541–56. doi: 10.1002/ece3.1604 (PMC4567860; doi:10.1002/ece3.1604)
Supplement: Table S1. — Plot data. Table S2. Tree species data. [file ece30005-3541-sd1.docx]

Supplementary material

**Table S1 Plot data**

Summary of environment, community weighted means (CWMs) and functional diversity (FD) across all 231 plots used in the analysis.

| Variable | mean | sd | min | max |
| --- | --- | --- | --- | --- |
| ALTITUDE | 201.61 | 38.95 | 104.10 | 291.29 |
| SLOPE | 31.54 | 5.52 | 16.18 | 45.86 |
| SOLAR | 1300787.88 | 120478.63 | 1006441.31 | 1544914.37 |
| CURV_PR | -12.18 | 37.76 | -126.21 | 129.89 |
| CURV_PL | 5.89 | 42.98 | -159.97 | 122.40 |
| pHH2O | 4.81 | 0.24 | 4.19 | 5.60 |
| pHKCL | 3.79 | 0.15 | 3.46 | 4.39 |
| N | 0.26 | 0.03 | 0.10 | 0.36 |
| C | 3.95 | 0.71 | 0.93 | 6.51 |
| CN | 15.25 | 1.97 | 9.67 | 21.74 |
| ASPECT.N | -0.32 | 0.64 | -1.00 | 1.00 |
| ASPECT.E | -0.43 | 0.55 | -1.00 | 0.99 |
| CD.growth | 36.13 | 18.70 | -13.25 | 105.04 |
| FD_PSI50 | 0.06 | 0.06 | 0.00 | 0.23 |
| FD_HYDCOND | 0.04 | 0.05 | 0.00 | 0.22 |
| FD_B | 0.05 | 0.06 | 0.00 | 0.24 |
| FD_CONMEAN | 0.05 | 0.05 | 0.00 | 0.19 |
| FD_CONMAX | 0.05 | 0.05 | 0.00 | 0.20 |
| FD_VPDMAX | 0.05 | 0.05 | 0.00 | 0.19 |
| FD_CONMAXFIT | 0.05 | 0.06 | 0.00 | 0.19 |
| FD_CONMAXFITA | 0.04 | 0.05 | 0.00 | 0.19 |
| FD_VPDMAXFIT | 0.03 | 0.04 | 0.00 | 0.19 |
| FD_VPDPOI | 0.03 | 0.05 | 0.00 | 0.23 |
| FD_WOODDENS | 0.05 | 0.05 | 0.00 | 0.16 |
| FD_WPOT | 0.06 | 0.07 | 0.00 | 0.22 |
| FD_LA | 0.05 | 0.07 | 0.00 | 0.24 |
| FD_LDMC | 0.05 | 0.06 | 0.00 | 0.18 |
| FD_SLA | 0.04 | 0.05 | 0.00 | 0.14 |
| FD_LEAFT | 0.07 | 0.07 | 0.00 | 0.25 |
| FD_STOMDENS | 0.06 | 0.06 | 0.00 | 0.17 |
| FD_STOMSIZE | 0.04 | 0.05 | 0.00 | 0.19 |
| FD_STOIND | 0.04 | 0.05 | 0.00 | 0.17 |
| FD_LNC | 0.04 | 0.06 | 0.00 | 0.25 |
| FD_LCC | 0.03 | 0.04 | 0.00 | 0.17 |
| FD_CN | 0.05 | 0.06 | 0.00 | 0.25 |
| FD_CA | 0.04 | 0.05 | 0.00 | 0.16 |
| FD_K | 0.03 | 0.03 | 0.00 | 0.13 |
| FD_MG | 0.05 | 0.06 | 0.00 | 0.23 |
| FD_DIAMVEIN1 | 0.04 | 0.05 | 0.00 | 0.19 |
| FD_DIAMVEIN2 | 0.06 | 0.07 | 0.00 | 0.19 |
| FD_VEINLENGTH | 0.04 | 0.05 | 0.00 | 0.22 |
| FD_UPPEREPI | 0.06 | 0.06 | 0.00 | 0.23 |
| FD_PALIS | 0.06 | 0.06 | 0.00 | 0.17 |
| FD_SPONGY | 0.03 | 0.03 | 0.00 | 0.11 |
| FD_LOG10RATIO | 0.05 | 0.06 | 0.00 | 0.25 |
| FD_LEAFTHICK | 0.04 | 0.04 | 0.00 | 0.14 |
| FD_SUBEPID | 0.06 | 0.09 | 0.00 | 0.25 |
| FD_EPICELLSIZ | 0.05 | 0.08 | 0.00 | 0.25 |
| FD_PALSTR | 0.08 | 0.09 | 0.00 | 0.25 |
| FD_EXCRET | 0.01 | 0.04 | 0.00 | 0.25 |
| FD_DENSINTCEL | 0.08 | 0.08 | 0.00 | 0.25 |
| FD_COLSCLER | 0.02 | 0.05 | 0.00 | 0.25 |
| FD_PAPILL | 0.07 | 0.10 | 0.00 | 0.25 |
| FD_EXTRAFLORAL | 0.03 | 0.07 | 0.00 | 0.25 |
| CWM_PSI50 | -4.11 | 0.99 | -6.60 | -1.28 |
| CWM_HYDCOND | 3.32 | 2.60 | 0.64 | 17.52 |
| CWM_B | 1.81 | 1.29 | 0.32 | 6.90 |
| CWM_CONMEAN | 719.39 | 145.99 | 382.54 | 1324.24 |
| CWM_CONMAX | 2081.00 | 402.69 | 850.30 | 2948.90 |
| CWM_VPDMAX | 22.59 | 2.80 | 11.08 | 28.64 |
| CWM_CONMAXFIT | 0.43 | 0.07 | 0.28 | 0.67 |
| CWM_CONMAXFITA | 895.48 | 234.65 | 426.05 | 1915.99 |
| CWM_VPDMAXFIT | 24.87 | 9.71 | 6.17 | 73.81 |
| CWM_VPDPOI | 49.24 | 16.77 | 32.42 | 137.43 |
| CWM_WOODDENS | 0.57 | 0.06 | 0.39 | 0.75 |
| CWM_WPOT | -1.41 | 0.42 | -2.49 | -0.40 |
| CWM_LA | 10183.45 | 10716.38 | 881.83 | 51195.69 |
| CWM_LDMC | 466.68 | 49.79 | 338.98 | 595.59 |
| CWM_SLA | 11.37 | 1.41 | 8.23 | 15.36 |
| CWM_LEAFT | 0.47 | 0.18 | 0.19 | 0.89 |
| CWM_STOMDENS | 631.01 | 219.29 | 205.41 | 1204.37 |
| CWM_STOMSIZE | 58.36 | 5.56 | 38.32 | 68.56 |
| CWM_STOIND | 0.04 | 0.01 | 0.01 | 0.07 |
| CWM_LNC | 1.62 | 0.23 | 1.19 | 2.83 |
| CWM_LCC | 46.59 | 1.34 | 41.74 | 49.05 |
| CWM_CN | 30.21 | 4.18 | 16.37 | 40.08 |
| CWM_CA | 42.46 | 6.37 | 27.08 | 59.07 |
| CWM_K | 12.48 | 2.96 | 7.47 | 36.02 |
| CWM_MG | 4.66 | 1.62 | 2.32 | 8.82 |
| CWM_DIAMVEIN1 | 0.02 | 0.00 | 0.01 | 0.03 |
| CWM_DIAMVEIN2 | 0.01 | 0.00 | 0.00 | 0.01 |
| CWM_VEINLENGTH | 4.38 | 0.71 | 2.85 | 6.39 |
| CWM_UPPEREPI | 12.21 | 2.93 | 7.27 | 21.57 |
| CWM_PALIS | 69.91 | 12.90 | 32.43 | 103.12 |
| CWM_SPONGY | 65.20 | 14.28 | 31.58 | 158.86 |
| CWM_LOG10RATIO | 0.06 | 0.08 | -0.11 | 0.42 |
| CWM_LEAFTHICK | 164.76 | 29.35 | 105.23 | 286.91 |
| CWM_SUBEPID | 0.16 | 0.27 | 0.00 | 1.00 |
| CWM_EPICELLSIZ | 0.73 | 0.44 | -1.00 | 1.00 |
| CWM_PALSTR | 1.57 | 0.53 | 1.00 | 2.50 |
| CWM_EXCRET | 0.02 | 0.11 | 0.00 | 1.00 |
| CWM_DENSINTCEL | 0.45 | 0.28 | 0.00 | 1.00 |
| CWM_COLSCLER | 0.06 | 0.19 | 0.00 | 1.00 |
| CWM_PAPILL | 0.23 | 0.33 | 0.00 | 1.00 |
| CWM_EXTRAFLORAL | 0.09 | 0.23 | 0.00 | 1.00 |

**Table S2 Tree species data**

Tree species of site A of the BEF-China experiment and included in this study. Species names are in accordance with nomenclature in The Flora of China (http:// flora.huh.harvard.edu/china); d: deciduous, e: evergreen.

| Species name | Acer davidii Franch. | Castanopsis eyrei (Champion ex Bentham) Tutcher | Castanea henryi (Skan) Rehd. et Wils. | Castanopsis sclerophylla (Lindley & Paxton) Schottky | Choerospondias axillaris (Roxb.) Burtt et Hill | Cinnamomum camphora (Linn.) Presl | Cyclobalanopsis glauca (Thunberg) Oersted | Cyclobalanopsis myrsinifolia (Blume) Oersted | Daphniphyllum oldhamii (Hemsl.) Rosenthal | Diospyros japonica Siebold & Zuccarini | Koelreuteria bipinnata Franch. | Liquidambar formosana Hance |
| --- | --- | --- | --- | --- | --- | --- | --- | --- | --- | --- | --- | --- |
| Abbreviation | acedav | caseyr | cashen | casscl | choaxi | cincam | cycgla | cycmyr | dapold | diogla | koebip | liqfor |
| Family | Aceraceae | Fagaceae | Fagaceae | Fagaceae | Anacardiaceae | Lauraceae | Fagaceae | Fagaceae | Daphniphyllaceae | Ebenaceae | Sapindaceae | Altingiaceae |
| Leafhabit | d | e | d | e | d | e | e | e | e | d | d | d |
| PSI50 | -4.01 | -2.79 | -4.51 | -4.39 | -3.29 | -3 | -5.09 | -4.37 | -5.44 | -2.95 | -1.67 | -4.22 |
| Ks | 0.64 | 2.04 | 10.4 | 1.12 | 1.42 | 1.87 | 1.62 | 0.86 | 0.64 | 1.34 | 2.3 | 2.29 |
| B | 1.13 | 1.58 | 0.78 | 2.23 | 0.85 | 0.97 | 3.25 | 2.66 | 2.74 | 1.04 | 0.6 | 1.08 |
| CONMEAN | 325.11 | 908.2 | 778.36 | 848.52 | 683.03 | 976.58 | 671.11 | 382.54 | 702.9 | 806.52 | 575.65 | 518.06 |
| CONMAX | 1223 | 2177.7 | 1764.9 | 2400 | 2041.4 | 2948.9 | 2609.9 | 850.3 | 2468.7 | 2402.4 | 1503 | 2314.6 |
| VPDMAX | 24.42 | 22.93 | 27.77 | 26.76 | 26.02 | 24.08 | 19.34 | 20.38 | 15.82 | 11.08 | 24.54 | 17.69 |
| CONMAXFIT | 0.33 | 0.49 | 0.54 | 0.41 | 0.44 | 0.41 | 0.32 | 0.5 | 0.36 | 0.41 | 0.52 | 0.28 |
| CONMAXFITA | 408.64 | 1071.35 | 953.08 | 974.45 | 896.07 | 1222.02 | 837.99 | 426.05 | 878.52 | 987.9 | 780.68 | 655.46 |
| VPDMAXFIT | 15.74 | 25.48 | 23.56 | 32.49 | 22.65 | 23.94 | 24.96 | 6.17 | 22.63 | 18.8 | 31.74 | 17.89 |
| VPDPOI | 30.22 | 44.72 | 42.54 | 65.13 | 39.01 | 41.33 | 43.03 | 38.75 | 39.58 | 36.81 | 56.98 | 32.42 |
| WOODDENS | 0.5 | 0.54 | 0.61 | 0.55 | 0.61 | 0.54 | 0.55 | 0.56 | 0.45 | 0.56 | 0.52 | 0.51 |
| WPOT | -0.64 | -1.61 | -1.79 | -1.65 | -0.5 | -1.91 | -1.44 | -1.77 | -1 | -1.33 | -1.9 | -0.4 |
| LA | 4540.08 | 881.83 | 3128.78 | 1820.74 | 35484.89 | 2083.27 | 2474.86 | 1633.62 | 2795.09 | 6245.87 | 30727.09 | 5051.43 |
| LDMC | 356.69 | 497.53 | 486.08 | 500.52 | 429.46 | 439.47 | 488.11 | 504 | 342.96 | 368.05 | 498.49 | 421.23 |
| SLA | 17 | 9.35 | 11.83 | 8.23 | 11.57 | 11.03 | 8.35 | 10.34 | 10.67 | 15.36 | 13.66 | 12.51 |
| LEAFT | 0.22 | 0.77 | 0.34 | 0.75 | 0.2 | 0.56 | 0.77 | 0.57 | 0.62 | 0.24 | 0.19 | 0.3 |
| STOMDENS | 783.95 | 488.6 | 765.66 | 555.78 | 562.96 | 625.45 | 657.31 | 672.63 | 418.67 | 366.05 | 849.58 | 1052.81 |
| STOMSIZE | 31.87 | 57.2 | 64.39 | 59.19 | 56.49 | 67.08 | 58.23 | 61.6 | 67.77 | 58.44 | 47.02 | 45.52 |
| STOIND | 0.02 | 0.03 | 0.05 | 0.03 | 0.03 | 0.04 | 0.04 | 0.04 | 0.03 | 0.02 | 0.04 | 0.05 |
| LNC | 1.46 | 1.38 | 1.87 | 1.38 | 1.79 | 1.63 | 1.45 | 1.54 | 1.19 | 1.78 | 1.4 | 1.53 |
| LCC | 45.36 | 47.13 | 47.36 | 47.16 | 46.66 | 47.67 | 46.96 | 46.81 | 45.4 | 47.55 | 47.15 | 45.89 |
| CN | 31.32 | 34.79 | 25.39 | 36.99 | 26.88 | 29.61 | 32.71 | 31.05 | 40.08 | 26.96 | 34.83 | 30.4 |
| CA | 43.35 | 34.26 | 28.9 | 27.08 | 59.07 | 49.03 | 44.68 | 47.99 | 52.03 | 42.23 | 51.37 | 47.26 |
| K | 15.27 | 12.34 | 13.98 | 10.96 | 9.03 | 9.67 | 10.79 | 15.24 | 16.54 | 36.02 | 12.81 | 14.83 |
| MG | 5.32 | 3.03 | 2.97 | 2.32 | 8.82 | 4.5 | 2.71 | 4.13 | 6.83 | 6.69 | 5.82 | 5.83 |
| DIAMVEIN1 | 0.02 | 0.01 | 0.02 | 0.01 | 0.02 | 0.02 | 0.03 | 0.02 | 0.02 | 0.03 | 0.01 | 0.02 |
| DIAMVEIN2 | 0.01 | 0.01 | 0.01 | 0.01 | 0.01 | 0.01 | 0.01 | 0.01 | 0.01 | 0.01 | 0.01 | 0.01 |
| VEINLENGTH | 3.07 | 6 | 3.68 | 5.16 | 4.16 | 4.09 | 5.29 | 4.85 | 3.6 | 3.54 | 4.8 | 3.4 |
| UPPEREPI | 11.4 | 10.75 | 8.73 | 7.27 | 9.08 | 19.49 | 14.23 | 21.57 | 16.8 | 15.7 | 10.82 | 14.62 |
| PALIS | 70.09 | 103.12 | 57.98 | 93.59 | 73.58 | 52.14 | 80.08 | 90.05 | 97.5 | 92.49 | 77.93 | 81.86 |
| SPONGY | 69.43 | 122.1 | 71.53 | 78.19 | 55.5 | 63.7 | 77.64 | 73.2 | 158.86 | 71.34 | 65.75 | 53.38 |
| LOG10RATIO | 0.02 | -0.02 | -0.05 | 0.11 | 0.15 | -0.06 | 0.1 | 0.11 | -0.11 | 0.14 | 0.1 | 0.19 |
| LEAFTHICK | 167.14 | 262.13 | 147.43 | 207.02 | 132.78 | 157.77 | 192.31 | 226.96 | 286.91 | 201.97 | 160.69 | 156 |
| SUBEPID | 0 | 1 | 0 | 1 | 0 | 0 | 0 | 0 | 0 | 0 | 0 | 0 |
| EPICELLSIZ | 0 | 1 | 1 | 0 | 1 | 1 | 1 | 1 | 1 | -1 | 1 | 1 |
| PALSTR | 1 | 2.5 | 1 | 2.5 | 1 | 1.5 | 2.5 | 2.5 | 2 | 1 | 2.5 | 2 |
| EXCRET | 0 | 0 | 0 | 0 | 0 | 1 | 0 | 0 | 0 | 0 | 0 | 0 |
| DENSINTCEL | 0.5 | 0.5 | 0 | 1 | 1 | 0.5 | 0 | 0.5 | 0 | 0 | 0.5 | 0 |
| COLSCLER | 0 | 0 | 0 | 0 | 0 | 0 | 0 | 0 | 0 | 0 | 1 | 0 |
| PAPILL | 0 | 0 | 0 | 0 | 0 | 0 | 0 | 1 | 1 | 0 | 0 | 0 |
| EXTRAFLORAL | 0 | 0 | 0 | 0 | 0 | 0 | 0 | 0 | 0 | 1 | 0 | 0 |

**Table S2 continued**

| Species name | Lithocarpus glaber (Thunb.) Nakai | Melia azedarach Linn. | Nyssa sinensis Oliver | Quercus acutissima Carruthers | Quercus fabri Hance | Quercus serrata Murray | Rhus chinensis Mill. | Sapindus mukorossi Gaertn | Triadica cochinchinensis Loureiro | Triadica sebifera (L.) Small | Schima superba Gardn. et Champ. |
| --- | --- | --- | --- | --- | --- | --- | --- | --- | --- | --- | --- |
| Abbreviation | litgla | melaze | nyssin | Queacu | quefab | queser | rhuchi | sapmuk | sapdis | sapseb | schsup |
| Family | Fagaceae | Meliaceae | Nyssaceae | Fagaceae | Fagaceae | Fagaceae | Anacardiaceae | Sapindaceae | Euphorbiaceae | Euphorbiaceae | Theaceae |
| Leafhabit | e | d | d | D | d | d | d | d | d | d | e |
| PSI50 | -6.6 | -1.85 | -3.54 | -3.39 | -5.14 | -2.7 | -2.42 | -3.57 | -1.28 | -4.32 | -5.65 |
| Ks | 4.23 | 17.52 | 1.95 | 2.42 | 3.15 | 2.87 | 3.6 | 1.76 | 1.28 | 11.01 | 1.47 |
| B | 6.9 | 0.57 | 0.45 | 1.45 | 1.07 | 0.82 | 0.5 | 1.38 | 0.32 | 0.62 | 2.03 |
| CONMEAN | 581.72 | 800.94 | 783.63 | 1015 | 772.25 | 473.13 | 1128.18 | 766.08 | 1324.24 | 916.88 | 750.64 |
| CONMAX | 1839.1 | 1816.8 | 1819.2 | 2000.8 | 1708.4 | 1719.9 | 2859.4 | 2321.4 | 2558.6 | 2653 | 2319.3 |
| VPDMAX | 22.54 | 28.64 | 14.32 | 21.42 | 19.93 | 24.64 | 23.19 | 27.01 | 14.13 | 21.54 | 24.5 |
| CONMAXFIT | 0.35 | 0.39 | 0.49 | 0.58 | 0.55 | 0.33 | 0.67 | 0.43 | 0.64 | 0.45 | 0.38 |
| CONMAXFITA | 648.63 | 702.7 | 895.65 | 1151.9 | 945.6 | 559.86 | 1915.99 | 1008.09 | 1640.65 | 1190.47 | 881.64 |
| VPDMAXFIT | 19.23 | 9.18 | 20.48 | 17.22 | 21.18 | 20.51 | 73.81 | 23.53 | 28.74 | 20.24 | 32.38 |
| VPDPOI | 44.04 | 61.49 | 41.99 | 36.24 | 37.63 | 39.17 | 137.43 | 40.45 | 54.28 | 36.14 | 65.84 |
| WOODDENS | 0.49 | 0.47 | 0.51 | 0.75 | 0.67 | 0.69 | 0.45 | 0.73 | 0.39 | 0.5 | 0.56 |
| WPOT | -1.34 | -1.65 | -1.05 | -2.08 | -1.84 | -2.49 | -0.4 | -1.75 | -1.43 | -1.1 | -1.12 |
| LA | 1956.09 | 51195.69 | 5866.24 | 2930.25 | 1912.07 | 1972.78 | 26235.35 | 42231.36 | 3018.95 | 2108.5 | 3230.61 |
| LDMC | 485.17 | 338.98 | 467.21 | 511.44 | 595.59 | 584.64 | 427.67 | 391.43 | 405.25 | 403.53 | 399.92 |
| SLA | 10.05 | 13.78 | 13.08 | 11.57 | 12.1 | 12.78 | 9.12 | 12.31 | 14.38 | 13.25 | 11.26 |
| LEAFT | 0.89 | 0.24 | 0.2 | 0.46 | 0.56 | 0.49 | 0.25 | 0.3 | 0.27 | 0.32 | 0.5 |
| STOMDENS | 290.96 | 572.09 | 344.34 | 1021.97 | 1154.55 | 1204.37 | 205.41 | 417.96 | 433.38 | 326.94 | 440.61 |
| STOMSIZE | 65.15 | 53.33 | 67.5 | 59.72 | 56.11 | 56.91 | 38.32 | 64.33 | 54.14 | 55.44 | 68.56 |
| STOIND | 0.02 | 0.03 | 0.02 | 0.06 | 0.06 | 0.07 | 0.01 | 0.03 | 0.02 | 0.02 | 0.03 |
| LNC | 1.36 | 2.83 | 1.31 | 1.99 | 1.96 | 1.62 | 1.76 | 1.89 | 1.87 | 2.09 | 1.36 |
| LCC | 48.11 | 46.12 | 45.52 | 48.43 | 47.05 | 42.08 | 49.05 | 46.19 | 45.79 | 43.87 | 48.01 |
| CN | 36.19 | 16.37 | 35.24 | 24.5 | 24.43 | 26.19 | 28.04 | 25.39 | 24.79 | 21.4 | 36.41 |
| CA | 38.98 | 53.84 | 38.44 | 36.33 | 43.27 | 40.28 | 48.35 | 46.9 | 42.57 | 46.89 | 33.09 |
| K | 7.47 | 22.48 | 12.98 | 9.88 | 11.16 | 8.54 | 13.47 | 15.23 | 9.49 | 9.56 | 15.8 |
| MG | 2.8 | 4.91 | 7.28 | 2.43 | 2.7 | 2.64 | 6.52 | 7.47 | 5.05 | 6.82 | 4.48 |
| DIAMVEIN1 | 0.02 | 0.01 | 0.01 | 0.02 | 0.02 | 0.01 | 0.02 | 0.03 | 0.02 | 0.02 | 0.01 |
| DIAMVEIN2 | 0.01 | 0 | 0.01 | 0.01 | 0.01 | 0.01 | 0.01 | 0.01 | 0.01 | 0.01 | 0.01 |
| VEINLENGTH | 3.24 | 3.67 | 4.87 | 6.21 | 6.39 | 2.85 | 3.77 | 4.65 | 4.01 | 3.96 | 4.62 |
| UPPEREPI | 8.15 | 11.28 | 10.88 | 12.03 | 12.58 | 11.99 | 20.32 | 10.84 | 8.86 | 11.74 | 11.08 |
| PALIS | 70.73 | 58.86 | 64.65 | 53.27 | 66.87 | 46.16 | 78.46 | 32.43 | 53.18 | 87.75 | 53.91 |
| SPONGY | 84.12 | 56.34 | 70.92 | 44.16 | 58.19 | 47.58 | 31.58 | 39.35 | 43.45 | 60.45 | 73.48 |
| LOG10RATIO | -0.05 | 0.04 | 0.04 | 0.09 | 0.11 | 0.01 | 0.42 | -0.04 | 0.1 | 0.17 | -0.11 |
| LEAFTHICK | 197.75 | 129.91 | 161.05 | 126.91 | 151.75 | 114.72 | 141.33 | 105.23 | 114.96 | 175.95 | 160.81 |
| SUBEPID | 1 | 0 | 0 | 0 | 0 | 0 | 0 | 0 | 0 | 0 | 0 |
| EPICELLSIZ | -1 | 0 | 1 | 1 | 1 | 1 | 1 | 1 | 1 | 1 | 1 |
| PALSTR | 1.5 | 1 | 1 | 1 | 1 | 2 | 1 | 1 | 1 | 1 | 1 |
| EXCRET | 0 | 0 | 0 | 0 | 0 | 0 | 0 | 0 | 0 | 0 | 0 |
| DENSINTCEL | 0 | 0 | 0.5 | 0.5 | 0.5 | 0.5 | 1 | 0.5 | 0.5 | 1 | 0.5 |
| COLSCLER | 0 | 0 | 0 | 0 | 0 | 0 | 0 | 0 | 0 | 0 | 0 |
| PAPILL | 1 | 0 | 0 | 0 | 0 | 0 | 1 | 0 | 0 | 1 | 0 |
| EXTRAFLORAL | 0 | 1 | 0 | 0 | 0 | 0 | 0 | 0 | 1 | 1 | 0 |
