# Supplementary material for: Early subtropical forest growth is driven by community mean trait values and functional diversity rather than the abiotic environment
Source: Ecol Evol. 2015 Aug 6;5(17):3541–56. doi: 10.1002/ece3.1604 (PMC4567860; doi:10.1002/ece3.1604)
Supplement: Table S3. — Principal Component Scores. [file ece30005-3541-sd2.docx]

**Table S3 Principal Component Scores**

Principal Component Scores for the species data used in the analysis including the first three dimensions. For species abbreviations see Table S2.

| Species | 1 | 2 | 3 |
| --- | --- | --- | --- |
| acedav | 0.06 | 0.01 | -0.11 |
| caseyr | -0.20 | 0.02 | 0.04 |
| cashen | 0.02 | -0.10 | -0.04 |
| casscl | -0.21 | 0.00 | 0.04 |
| choaxi | 0.12 | -0.03 | 0.04 |
| cincam | -0.01 | -0.04 | 0.04 |
| cycgla | -0.13 | 0.02 | 0.00 |
| cycmyr | -0.13 | 0.05 | 0.01 |
| dapold | -0.10 | 0.21 | -0.03 |
| diogla | 0.14 | 0.13 | -0.11 |
| koebip | -0.01 | -0.07 | -0.01 |
| liqfor | 0.00 | 0.02 | -0.08 |
| litgla | -0.19 | 0.16 | -0.13 |
| melaze | 0.20 | -0.02 | -0.19 |
| nyssin | 0.03 | -0.01 | 0.01 |
| queacu | 0.00 | -0.17 | 0.04 |
| quefab | -0.02 | -0.14 | 0.04 |
| queser | -0.06 | -0.18 | -0.07 |
| rhuchi | 0.17 | 0.15 | 0.26 |
| sapmuk | 0.11 | -0.08 | -0.02 |
| sapdis | 0.19 | 0.02 | 0.06 |
| sapseb | 0.14 | 0.12 | 0.03 |
| schsup | -0.03 | -0.01 | 0.00 |
